# Invisible transportation infrastructure technology to mitigate energy and environment

**DOI:** 10.1186/s13705-017-0128-x

**Published:** 2017-09-11

**Authors:** Md. Faruque Hossain

**Affiliations:** 1 Green Globe Technology, 4323 Colden Street, Suite 15L, Flushing, NY 11355 USA; 2 Department of Civil and Urban Engineering, New York University, 6 Metro Tech Center, Brooklyn, NY 11201 USA

**Keywords:** Maglev technology, Flying transportation, Wind energy for vehicle, Cost reduction, Transportation innovation

## Abstract

**Background:**

Traditional transportation infrastructure built by heat trapping products and the transportation vehiles run by fossil fuel, both causing deadly climate change. Thus, a new technology of invisible *Flying Transportation* system has been proposed to mitigate energy and environmental crisis caused by traditional infrastructure system.

**Methods:**

Underground *Maglev* system has been modeled to be constructed for all transportation systems to run the vehicle smoothly just over two feet over the earth surface by propulsive and impulsive force at flying stage. A wind energy modeling has also been added to meet the vehicle’s energy demand when it runs on a non-maglev area. Naturally, all maglev infrastructures network to be covered by evergreen herb except pedestrian walkways to absorb CO_2_, ambient heat, and moisture (vapor) from the surrounding environment to make it cool.

**Results:**

The research revealed that the vehicle will not require any energy since it will run by superconducting electromagnetic force while it runs on a maglev infrastructure area and directed by wind energy while it runs on non-maglev area.

**Conclusions:**

The proposed maglev transportation infrastructure technology will indeed be an innovative discovery in modern engineering science which will reduce fossil fuel energy consumption and climate change dramatically.

## Background

Urban and sub-urban area massively depends on transportation infrastructure networks which are primarily constructional with concrete and asphalt, and it does not have enough vegetation to absorb heat caused by these asphalt and concrete [1]. Recent research found that transportation infrastructure on earth is approximately 0.9% of the total planetary surface area of 196.9 million mi^2^ which is equivalent to 1.77 million mi^2^ infrastructure on earth which causes nearly 6% of global warming by reflecting heat (albedo) back to the space [2, 3]. On the other hand, conventional energy utilization for the transportation sectors is not only costly but also causing adverse environmental impact [4, 5]. A variety of studies have been performed to understand long-term climate variations by conventional energy utilization by the transportation sectors that is casing nearly 28% of global energy consumption which is equivalent to mega ton CO_2_ and is responsible for 28% percent of global warming, and thus infrastructure and transportation fuel cases total 34% global warming [6, 7]. In order to mitigate transportation infrastructure crisis and its adverse environmental impact, I, therefore, propose a new technology of maglev transportation infrastructure system for building better transportation infrastructure system.

A recent study by Cai and Chen described the dynamic characteristics, magnetic suspension systems, vehicle stability, and suspension control laws of maglev/guideway coupling systems about the maglev transportation system [8, 9], but that fact commercial application of this research modeling considering life cycle cost analysis, technology implementation and infrastructure development did not show the any possibility to apply it commercially [1, 10]. Therefore, the approach of this research is to apply the maglev transportation infrastructure commercially for confirming a greener and cleaner transportation infrastructure system where all vehicles shall run just over 2 ft above the earth surface at flying stage by the act of propulsive and impulsive superconducting force. Since the vehicle will run by electromagnetic force, it will not require any energy while running over the maglev. To mitigate energy consumption when the vehicle needs to run on a maglev area, additional technology has also been proposed to implement wind energy into the vehicle while it is in motion as a backup energy source. Thus, a detailed mathematical modeling using Matlab Simulink software has been implemented for this wind energy utilization for the vehicles by performing turbine and drivetrain modeling [11–13]. A concerted research effort has been performed recently on climate science and found that currently 400 ppm CO_2_ is present in the atmosphere causing global warming, which required to cut down 300 ppm CO_2_ to confirm global cooling at comfortable stage [14–16]. Once maglev transportation infrastructure system is implemented throughout the world, it will reduce 34% of CO_2_ per year. Thus, it will take only $\left\{{\int}_{300}^{402}\left(1-0.34\right) dx\right\}=66$ years to cool the atmosphere, resulting no more climate change after 66 years. Simply, it will be the most innovative technology in modern science to mitigate the cost and global warming dramatically.

## Simulations and methods

In order to present maglev transportation infrastructure modeling, I have formulated the following calculation by using Matlab software in terms of (1) guideway model system by adopting Bernoulli-Euler beam equation of series of simply supported beams; (2) Calculation of magnetic forces for uplift levitation and lateral guidance with allowable levitation and guidance distance considering lateral vibration control LQR algorithm, tuning parameters, and Maglev Dynamics.

## Guideway model

**Fig. 1:**
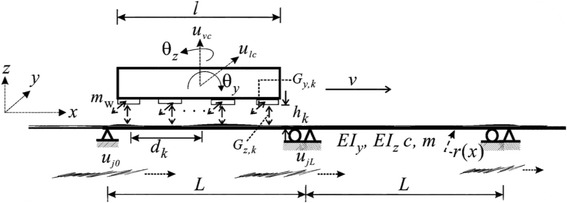
A free body diagram shows the maglev guideway vs vehicle force considering weight and motion where the superconducting guideway is below the vehicle body. It is functioned by series of equal-distant concentrated masses to levitate the vehicle up to the superconducting guideway beam; the maglev bar gets stimulated by the lateral multi-support motion which is induced by the superconducting force to allow traveling on longitudinal direction

To prepare the guideway modeling considering free body diagram (Fig. 1), I have considered multiple magnets with equal intervals (*d*) that is to be traveling at a various level speeds of speed *v*, where *m* = beam weight, *c* = damping coefficient, EI_*y*_ = flexural rigidity in the *y* direction, EI_*z*_ = flexural rigidity in the *z* direction, *l* = car length, *m*w = lumped mass of magnetic wheel, *mv* = distributed mass of the rigid car body, and ${\theta}_{i_{=\mathrm{x},\mathrm{y},\mathrm{z}}}=$ midpoint rotation components of the rigid car body. Considering these, I have formulated the equations of motion for the *j*th guideway girder carrying a moving maglev vehicle suspended by multiple magnetic forces as follows:

1 $$ m{\ddot{u}}_{y,j}+{c}_y{\dot{u}}_{y,j}+{EI}_y{u}_{y,j}^{""}={\sum}_{k=1}^K\left[{G}_{y,k}\left({i}_k,{h}_{y,k}\right){\varphi}_j\left({x}_k,t\right)\right] $$

2 $$ m{\ddot{u}}_{z,j}+{c}_z{\dot{u}}_{z,j}+{EI}_z{u}_{z,j}^{""}={p}_0-{\sum}_{k=1}^K\left[{G}_{z,k}\left({i}_k,{h}_{z,k}\right){\varphi}_j\left({x}_k,t\right)\right] $$

and

3 $$ {\varphi}_j\left({x}_k,t\right)=\delta \left(x-{x}_k\right)\left[H\left(t-{t}_k-\frac{\left(j-1\right)L}{v}\right)-H\left(t-{t}_k-\frac{jL}{v}\right)\right] $$

together with the following boundary conditions with lateral (*y* direction) support movements:

4 $$ {\displaystyle \begin{array}{l}{u}_{y,j}\left(0,t\right)={u}_{yj0}(t),{u}_{y,j}\left(L,t\right)={u}_{yj L}(t),\\ {}\kern1.8em {EI}_z{u}_{z,j}^{"}\left(0,t\right)={EI}_z{u}_{z,j}^{"}\left(L,t\right)=0\end{array}} $$

5 $$ {u}_{z,j}\left(0,t\right)={u}_{z,j}\left(L,t\right)=0 $$

$$ {EI}_y{u}_{y,j}^{"}\left(0,t\right)={EI}_y{u}_{y,j}^{"}\left(L,t\right)=0 $$

where (●)′ = ∂(●)/∂*x*, (●) = ∂(●)/∂*t*, *u*_*z,j*_(*x, t*) = vertical deflection of the *j*th span, *u*_*y,j*_(*x, t*) = lateral deflection of the *j*th span, *L* = span length, *K* = number of magnets attached to the rigid levitation frame, *δ* (●) = Dirac’s delta function, *H*(*t*) = unit step function, *k* = 1, 2, 3, …, *K*th moving magnetic wheel on the beam, *tk* = (*k−*1)*d/v* = arrival time of the *k*th magnetic wheel into the beam, *xk* = position of the *k*th magnetic wheel on the guideway, and (*G*_*y,k*_, *G*_*z,k*_) = lateral guidance and uplift levitation forces of the *k*th lumped magnet in the vertical and lateral directions [17, 18].

## Magnetic forces of uplift levitation and lateral guidance

Since the maglev vehicle will run over guideway by superconducting force with lateral ground motion (as shown in Fig. 1), guidance forces tuned by the maglev system need to be controlled by the lateral motion of the moving maglev vehicle. Therefore, this study adopts the lateral guidance force (*G*_*y,k*_) and the uplift levitation force (*G*_*z,k*_) [19, 20] to keep and guide the *k*th magnet of the vehicle, those could be expressed as:

6 $$ {G}_{y,k}={K}_0{\left(\frac{i_k(t)}{h_{z,k(t)}}\right)}^2{K}_{k,z} $$

7 $$ {G}_{y,k}={K}_0{\left(\frac{i_k(t)}{h_{z,k(t)}}\right)}^2\left(1-{K}_{y,k}\right) $$

where *Ky*,*k* and *Kz*,*k* represent induced guidance factors, and they are given by:

8 $$ {K}_{y,k}=\frac{X_k\ x\ {h}_{y,k}}{W\left(1+{X}_k\right)},{K}_{z,k}=\frac{X_k\ x\ {h}_{y,k}}{W\left(1+{X}_k\right)} $$

In Eqs. (6) and (7), *K*_0_ = *µ*_0_ *N*_0_^2^ *A*_0_/4 = coupling factor, *χ*_*k*_ = *π h*_*y,k*_/4*h*_*z,k*_, *W* = pole width, *µ*_0_ = vacuum permeability, *N*o = number of turns of the magnet windings, *A*o = pole face area, *i*_*n*_(*t*) = *i*_0_ + *ι*_*n*_ (*t*) = electric current, *ι*_*n*_ (*t*) = deviation of current, and (*i*_0_, *h*_y0_, *h*_z0_) = desired current and air gaps around a specified nominal operating point of the maglev wheels at *static* equilibrium. And the uplift levitation (*h*_*y,k*_) and lateral guidance (*h*_*z,k*_) gaps are respectively given by:

9 $$ {h}_{y,k}(t)={h}_{y0}+{u}_{l,k}(t)-{u}_{y,j}\left({x}_k\right),{u}_{l,k}(t)={u}_{lc}(t)+{d}_k{\theta}_z $$

10 $$ {h}_{z,k}(t)={h}_{z0}+{u}_{v,k}(t)-{u}_{z,j}\left({x}_k\right)+r\left({x}_k\right),{u}_{v,k}(t)={u}_{vc}(t)+{d}_k{\theta}_y $$

where (*u*_*l,k*_ *, u*_*v,k*_) = displacements of the *k*th magnetic wheel in the *y* and *z* directions, (*u*_*lc*_ *, u*_*vc*_) = midpoint displacements of the rigid car, (*θy*,*θz*) = midpoint rotations of the rigid car, *r*(*x*) = irregularity of guideway, and *dk* = location of the *k*th magnetic wheel to the midpoint of the rigid beam. As indicated in Eqs. (6)–(8), the motion-dependent nature and guidance factors (*Ky*,*k*, *Kz*,*k*) dominate the control forces of the maglev vehicle-guideway system. Next, the equations of motion of the 4-DOFs tigid maglev vehicle (see Fig. 1) are written as:

11 $$ {M}_0{\ddot{u}}_{lc}=g(t)+{\sum}_{k=1}^K{G}_{y,k},\kern0.5em {I}_T{\ddot{\theta}}_Z=g(t)\ \mathrm{x}\ l+{\sum}_{k=1}^K\left[{G}_{y,k}{d}_k\right] $$

12 $$ {M}_0{\ddot{u}}_{vc}={p}_0+{\sum}_{k=1}^K{G}_{z,k},\kern0.5em {I}_T{\ddot{\theta}}_y=-{\sum}_{k=1}^K\left[{G}_{z,k}{d}_k\right] $$

in which *M*_0_ = *m*_*v*_ *l + Km*_*w*_ = lumped mass of the vehicle, *g*(*t*) = control force to tune the lateral response of the maglev vehicle, *I*_*T*_ = total mass moment of inertia of the rigid car, and *p*_0_ = *M*_0_ *g* = lumped weight of the maglev vehicle.

## Wind energy modeling for the vehicles

Though the vehicle will run by electromagnetic force, a wind turbine generator is to be used for powering vehicle as the additional source of energy to exit vehicle from road and park where maglev system is not available. Thus, the model is developed by doubly fed induction generator (DFIG) for producing electricity for transportation vehicles [21]. The fundamental equation governing the mechanical power of the wind turbine is

13 $$ {P}_w=\frac{1}{2}{C}_p\left(\lambda, \beta \right)\rho {AV}^3 $$

where *ρ* is the air density (kg/m^3^), *C*_*p*_ is the power coefficient, *A* is the intercepting area of the rotor blades (m^2^), *V* is the average wind speed (m/s), and *λ* is the tip speed ratio [16]. The theoretical maximum value of the power coefficient *C*_*p*_ is 0.593; *C*_*p*_ is also known as Betz’s coefficient. Mathematically,

14 $$ \lambda =\frac{R\omega}{V} $$

where *R* is the radius of the turbine (m), *ω* is the angular speed (rad/s), and *V* is the average wind speed (m/s). The energy generated by wind can be obtained by

15 $$ {Q}_w=P\times (Time)\left[\mathrm{kWh}\right] $$

It is well known that wind velocity cannot be obtained by a direct measurement from any particular motion [22, 23]. In data taken from any reference, the motion needs to be determined for that particular motion; then, the velocity needs to be measured at a lower motion.

16 $$ v(z)\ln \left(\frac{z_r}{z_o}\right)=v\left({z}_r\right)\ln \left(\frac{z}{z_0}\right) $$

where *Z*_*r*_ is the reference height (m), *Z* is the height at which the wind speed is to be determined, *Z*_*0*_ is the measure of surface roughness (0.1–0.25 for crop land), *v*(*z*) is the wind speed at height z (m/s), and *v*(*z*_*r*_) is the wind speed at the reference height *z* (m/s). The power output in terms of the wind speed shall be estimated using the following equation:

17 $$ {P}_w(v)=\left\{\begin{array}{l}\frac{v^k-{v}_C^k}{v_R^k-{v}_C^k}\cdot {P}_R\kern1.08em {v}_C\le v\le {v}_R\\ {}{P}_R\kern3.359999em {v}_R\le v\le {v}_F\\ {}0\kern2.28em v\le {v}_C\;\mathrm{and}\;v\ge {v}_F\end{array}\right. $$

where *P*_*R*_ is rated power, *v*_*C*_ is the cut-in wind speed, *v*_*R*_ is the rated wind speed, *v*_*F*_ is the rated cut-out speed, and *k* is the Weibull shape factor [24]. When the blade pitch angle is zero, the power coefficient is maximized for an optimal TSR [2]. The optimal rotor speed is to be calculated by

18 $$ {\omega}_{opt}=\frac{\lambda_{opt}}{R}{V}_{wn} $$

which will give

19 $$ {V}_{wn}=\frac{R{\omega}_{opt}}{\lambda_{opt}} $$

where *ω*_opt_ is the optimal rotor angular speed in rad/s, *λ*_*opt*_ is the optimal tip speed ratio, *R* is the radius of the turbine in meters, and *V*_*wn*_ is the wind speed in m/s.

**Fig. 2:**
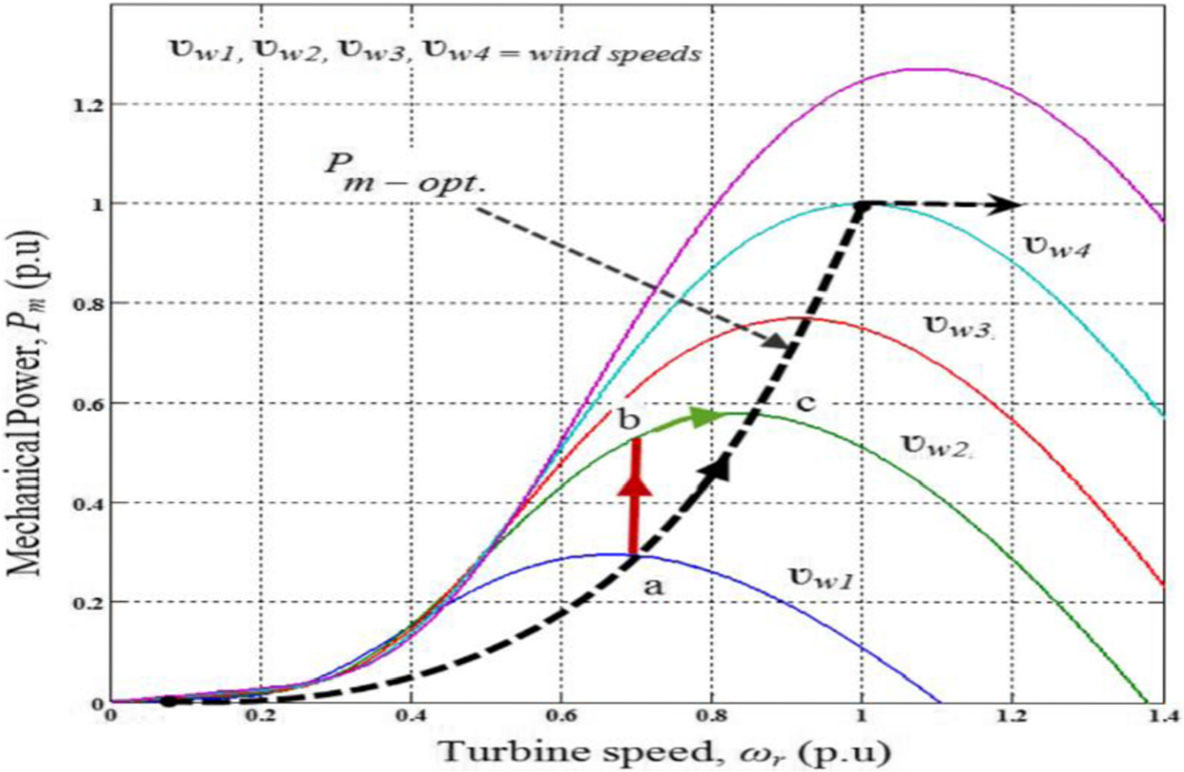
Relationship between mechanical power generation and turbine speeds at different wind speeds for an implementation in a car

The turbine speed and mechanical powers are depicted in the following graph (Fig. 2), with increasing and decreasing rates of wind speed while the vehicle is in motion [25, 26]. When the wind is steady, the persistence forecasts yield good results [27, 28]. When the wind speed is increased rapidly, sudden “ramps” in power output are generated, which is a tremendous benefit for capturing the energy.

## Wind energy storage in battery system

Standard Simulink/Sim Power Systems has been calculated by using Matlab-Simulink for the wind energy conversion that is to be stored in circuit-implemented inverter as a storage buffer, and all the electricity is to be supplied through the battery according to Peukert’s law to start the engine and to be used when the vehicle is not in motion [19, 24, 29].

## Design of traffic control

**Fig. 3:**
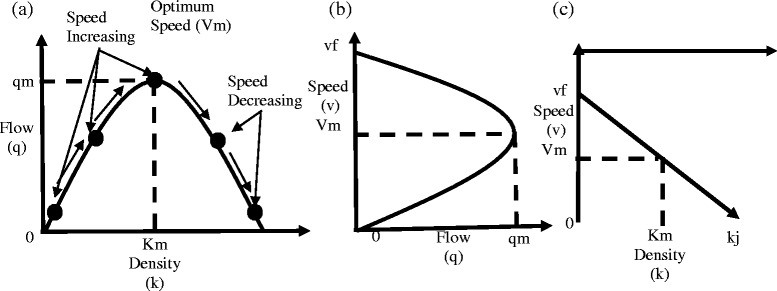
The Greenshield’s Fundamental Diagrams. **a** Speed vs vehicle density. **b** flow vs vehicle density, and **c** speed vs flow analysis

Though underground maglev system has the capability to allow run up to 580 kph, the vehicles’ high speed shall be calculated based on traffic flow, composition, volume, number and location of access points, and local environment importantly allotting sufficient number of lanes considering Greenshield’s following road and highway capacity analysis (Fig. 3).

Since the maglev technology is invisible, thus, to alert the drivers and pedestrian, the maglev roads, highways, and its exits should be constructed by landscaping by covering the guideway by herb (green grass) and in between lanes at least two feet to be left blank (no landscaping) in order to differentiate the lanes.

## Results and discussion

Based on the mathematical modeling described above, I have performed load resistant factor design (LRFD) calculation considering the following equation and selected W24 × 84 beam which is the continuous maglev underground runs (metal track guideway) that need to be structurally sound to carry enough current, load, and levitate force of the vehicles.

20 $$ Fy\propto \frac{n{l}^2}{h} $$

21 $$ Fx\propto \kern0.5em \frac{-1}{ktvx}\kern1.25em Fx\propto \frac{-n{l}^2}{h} $$

where Fy is the vehicle weight, *n* is the total number of coils in maglev, *l* is the current on each coil, *h* is the height of levitation, *t* is the thickness of conduction track, and *k* is the conductivity of track.

**Fig. 4:**
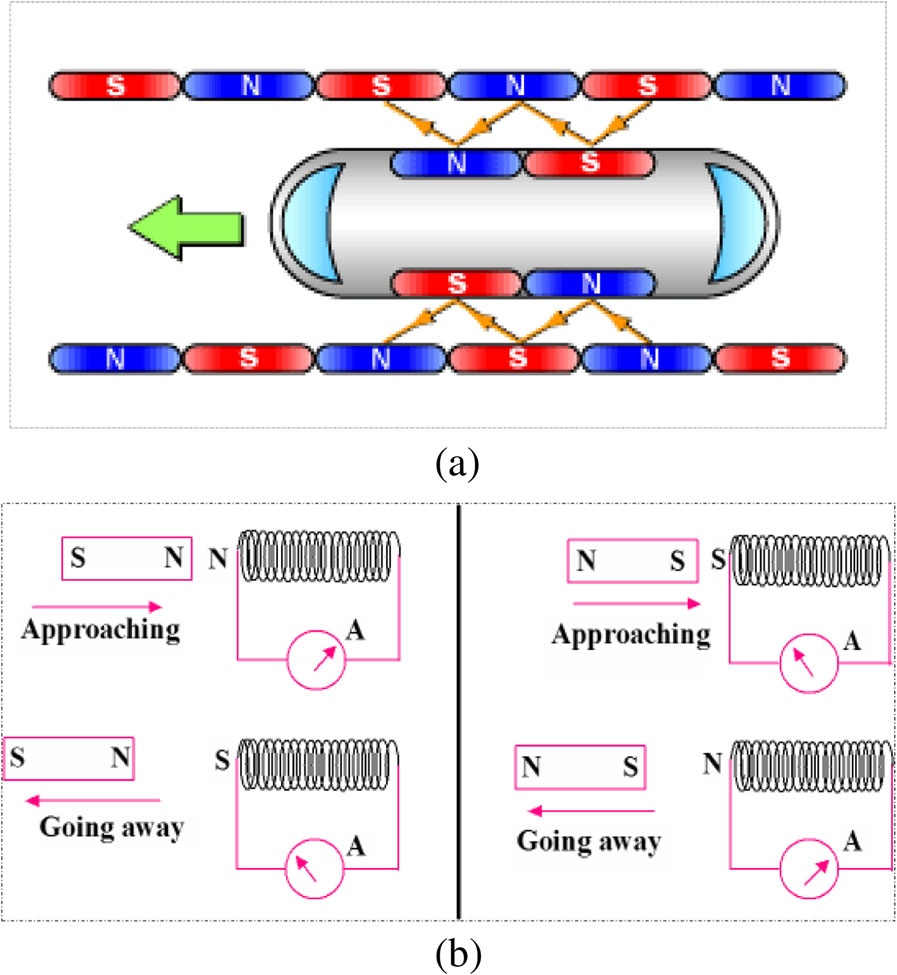
The above figures indicate the polarization of the coil in different cases: **a** Schematic diagram of the director of the running vehicle (must be construction with magnet as shown on this diagram) on maglev propulsion via propulsion coils. **b** Near the receding S-pole, becomes an N-pole to oppose the going away of the bar magnet’s S-pole

To construct under maglev guideway just 2 ft below of the earth surface, it will need to have a U-shaped cross-section to fix the pole position [30]. Naturally, heavy duty waterproofing membrane is to be used to protect the maglev underground runs for avoiding floods and moisture. It is well researched that the propulsion coils run in elliptical loops along both walls of the guideway, generating magnetic force when electricity runs through them [4]. So, levitation and guidance coils that will be formed will create their own magnetic force once the applied superconducting magnets pass on it, where propulsion and levitation are the key factor to run the vehicle. In propulsion, as the direction of the current charges back and forth in the propulsion coils above the wall of the guideway, the north and south poles will reserve repeatedly and shall propel the vehicle by alternating force of attracting and repulsion (Fig. 4). In levitation, as the vehicle passes, an electric current is induced in the coil along the guideway and the vehicle will be levitated by the force of attraction, which will pull up on the magnet in the vehicle, as well as by repulsion, which will push up on the magnet [31].

**Fig. 5:**
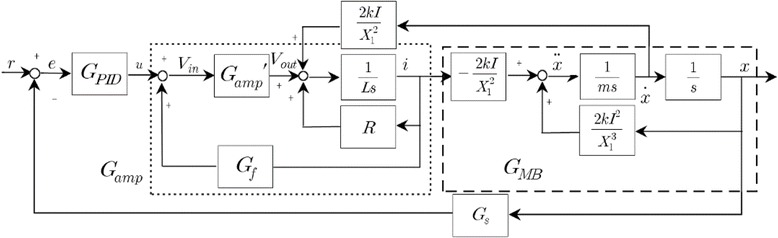
Block diagram to control the mathematically modeled magnetic bearing system, a process to design the driver to operate the electromagnet. Here, the method to determine the peripheral device values of the linear amplifier circuit that has the desired output by applying a generic algorithm and the method to identify the magnetic bearing system

To create levitation and lateral balance in the vehicle, an electromagnetic induction is to be used. To confirm the most efficient and economical way to produce a powerful magnetic field by using the superconducting coils, I have assumed the permanent currents of about 700,000 A go through these superconducting coils [31], hence creating a strong magnetic field of almost 5 T, i.e., 100,000 times stronger than the earth’s magnetic field by implementing the following block diagram (Fig. 5).

**Fig. 6:**
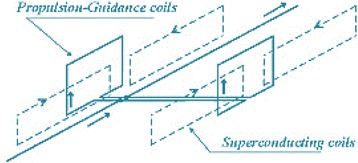
The Maglev vehicle’s force and directional diagram as shown by propulsion guidance coils and superconducting coils

Simply, it can be explained that when an electric current flows through the propulsion coils, a magnetic field is produced. The forces of attraction and repulsion between the coils and the superconducting magnets on the vehicle propel the vehicle forward in a flying stage up to 4 ft height where 2 ft shall be considered underground cover and the other 2 ft is just over the earth surface (Fig. 6). The vehicle’s speed is to be adjusted by altering the timing of the polarity shift in the propulsion coils’ magnetic field between north and south with the possibility of maximum speed of 580 kph [31]. As the vehicle passes just 2 ft above the guideway (1 ft from the earth surface), an electric current is induced in the levitation and guidance coils, creating opposite magnetic poles in the upper and lower loops. The upper loops become the polar opposite of the vehicle’s magnets, producing attraction, which pulls the vehicle up. The lower loops have the same pole as the magnets. This generates repulsion, which pushes the vehicle in the same direction up. The two forces combine to levitate the vehicle, while maintaining its lateral balance between the walls of the guideway.

Subsequently, a niobium-titanium alloy is to be used to create superconducting magnets for maglev, but to reach superconductivity, they must be kept cold. In order to keep the alloy cool, liquid helium should be used with a temperature of −269 °C since alloy retains superconductivity at temperatures up to −263 °C, though the maglev system can operate better at 6 °C to produce sufficient magnetic force.

In addition to underground maglev construction, the wind turbine generation system is to be installed on vehicles as the the backup energy source by the operational performace of wind turbine while vehicle is in a motion.

**Fig. 7:**
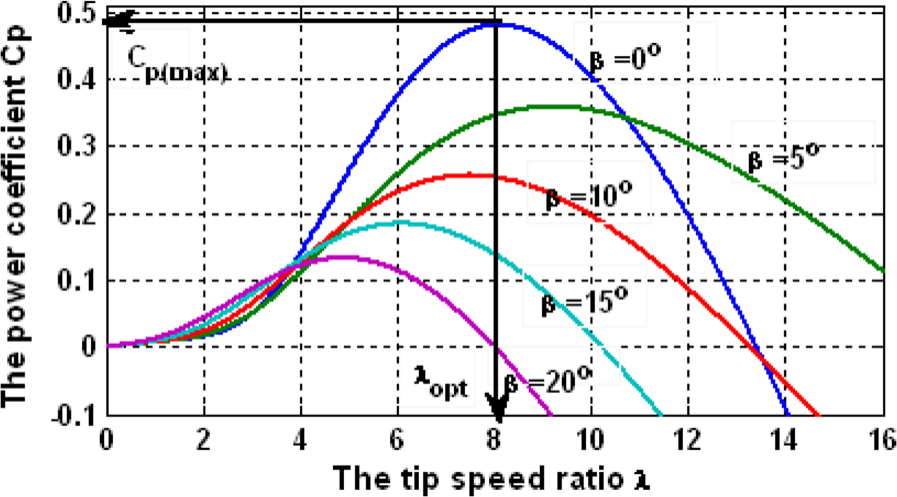
shows that the maximum values of *C*_*p*_ are achieved for the curve associated with *β* = 2°. From this curve, the maximum value of *C*_*p*_ (*C*_*p,max*_ = 0.5) is obtained for *λ*_*opt*_ = 0.91. This value (*λ*_*opt*_) represents the optimal speed ratio

These conditions permit application of the wind profile which is considered to be a wind speed signal with a mean value of 8 m/s and a rated wind speed of 10 m/s; the whole system is tested under standard conditions with a stator voltage of approximately 50% for 0.5 s between 4 and 4.5 s, approximately 25% between 6 and 6.5 s, and 50% between 8 and 8.5 s (Fig. 7). Thus, the machine is considered to be functioning in ideal conditions (no perturbations and no parameter variations). Moreover, to guarantee a unity power factor at the stator side, the reference for the reactive power is to be set to zero [32]. As a result of increasing wind speed, the generator shaft speed achieved maximum angular speed by tracking the maximum power point speed. Thus, the wind turbine always works optimally since the pole placement technique is to be used to design the tracking control [15]. Consequently, decoupling among the components of the rotor current was also performed to confirm that the control system worked effectively. The bidirectional active and reactive power transfer between the rotor and power system is exchanged by the generator according to the super synchronous operation, achieving the nominal stator power, and the reactive power can be controlled by the load side converter to obtain the unit’s power factor to generate energy for powering vehicles [6, 14].

## Construction cost estimate comparison

Order of magnitude cost estimate was performed by using HCSS (Heavy Bid) software standard union rate of New York State locals with a project of 10% general condition, 10% overhead and profit, and 3% contingency over the hard cost of labor, materials, and equipment comparing between maglev infrastructure and tradition infrastructure system for a sample of 100 miles long and 128 ft wide (12 ft wide of four lanes on each directions, two-sided 10 ft service space, and 6 ft median in the center of the road). In order to determine that the underground guideway (w24 × 84) can last long, I have calculated again the LRFD to provide the shoring of both sides for the entire 100 miles long and 128 ft wide (12 ft wide of 4 lane each directions two sided 10 ft service space and 6 ft median in the center of the road) construction cost considering standard excavation up to 6 ft deep, with appropriate shoring with minimum embedment depth L4 is 5 ft and standard soil pressure ϒ_s_ = 120 lbf/ft^3^, angle of pressure *Φ* = 21^0^, and the soil pressure coefficient *c* = 800 lbf/ft^2^. To prepare the conceptual estimate, we need to determine the length of soldier piles. I have counted 6′ OC (on center) soldier piles at both sides by illustrating and using the following LRFD method that soldier piles must be set at to support the necessary excavation and/or earth pressure against collapse.

22 $$ \mathrm{Active}\  \mathrm{Earth}\  \mathrm{Pressure}\kern1em \mathrm{Ka}={tan}^2\left({45}^{\mathrm{o}}-\frac{\varPhi }{2}\right) $$

23 $$ \mathrm{Passive}\  \mathrm{Earth}\  \mathrm{Pressure}\kern0.75em \mathrm{Kb}={tan}^2\left({45}^{\mathrm{o}}+\frac{\varPhi }{2}\right) $$

Use Eqs. (22) and (23) to find the lateral earth pressure the solid piles must support.

$$ {\displaystyle \begin{array}{l}{\mathrm{P}}_{\mathrm{EM}}={\varUpsilon}_{\mathrm{s}}\mathrm{h}\ {\mathrm{k}}_{\mathrm{a},\mathrm{piles}}\\ {}\kern1.08em =\left(120\;\frac{lbf}{ft^3}\right)\cdot (6.0)\;{tan}^2\left({45}^{\mathrm{o}}-\frac{21^{\mathrm{o}}}{2}\right)\\ {}\kern1.08em =340.128\;\mathrm{lbf}/{\mathrm{ft}}^2\end{array}} $$

to determine the type of steel beams required for the soldier piles, we have taken the bending moments about the tributary area of the piles.

$$ {\displaystyle \begin{array}{cccc}\hfill \begin{array}{l}{}_{\mathrm{Soil}\  \mathrm{pile}\  \mathrm{spacing}=6\mathrm{ft}}\\ {}{\kern0.24em }_{\mathrm{Side}\  \mathrm{elevation}}\end{array}\hfill & \hfill M=\hfill & \hfill \hfill & \hfill {}_{\mathrm{M}=2040.768\ \mathrm{ft}\hbox{-} \mathrm{lbf}/\mathrm{ft}}\hfill \\ {}\hfill \hfill & \hfill \mathrm{M}=\left(\frac{\left(340.128\kern0.5em \frac{lb}{ft^2}\right)\left(6\ \mathrm{ft}\right)}{2}\right)\hfill & \hfill \left(\frac{6\ \mathrm{ft}}{3}\right)\hfill & \hfill =2040.768\ \mathrm{ft}\hbox{-} \mathrm{lbf}/\mathrm{ft}\hfill \\ {}\hfill \hfill & \hfill \hfill & \hfill \hfill & \hfill \hfill \\ {}\hfill \hfill & \hfill \hfill & \hfill \hfill & \hfill \hfill \end{array}}\kern1.32em $$

The moment is a distributed moment applied to the base of the tributary area of each soldier pile. Therefore, the moment is 2040.768 ft-lbf per foot. The total moment on the soldier pile (at the base) is

$$ {\displaystyle \begin{array}{l}{M}_0=M\left(6\ \mathrm{ft}\right)\\ {}\kern0.96em =\left(2040.768\ \frac{ft- lbf}{ft}\ \right)\;\left(6\ \mathrm{ft}\right)\\ {}\kern0.84em =12,244.61\ \mathrm{ft}\hbox{-} \mathrm{lbf}\end{array}} $$

Now,

$$ {\displaystyle \begin{array}{l}{\mathrm{Z}}_{\mathrm{req}}=\frac{M0}{\varPhi b\  Fy}=\frac{\kern0.5em \left(12\ \frac{in}{ft}\right)\left(12,244.61\  ft- lbf\right)}{(0.9)\left(50,000\kern0.5em \frac{lbf}{in^2}\right)}\\ {}\kern0.96em =3.27\;{\mathrm{in}}^3\end{array}} $$

From AISC tables, the soldier piles have been selected as W12 × 26, and the perpendicular support w8 × 12 members 6 ft long.

Then, we have determined the depth required below subgrade by calculating the passive earth pressure coefficient using Eq. (23)

$$ {\displaystyle \begin{array}{l}{K}_p={tan}^2\left(\left({45}^{{}^{\circ}}+\frac{\varPhi }{2}\right)\right.\\ {}\kern0.84em ={tan}^2\left({45}^{{}^{\circ}}+\frac{21^{{}^{\circ}}}{2}\right)\\ {}\kern0.84em =2.12\end{array}} $$

Then, we have calculated the active earth pressure coefficient using Eq. (22)

$$ {\displaystyle \begin{array}{l}{K}_a={tan}^2\left({45}^{{}^{\circ}}-\frac{\varPhi }{2}\right)\\ {}\kern0.84em ={tan}^2\left({45}^{{}^{\circ}}-\frac{21^{{}^{\circ}}}{2}\right)\\ {}\kern0.84em =0.4724\end{array}} $$

In order to determine the slopes of the excavation, depth is required. Since below the bottom of the excavation, both pressure are considered to be passive and have the same slope, the slope of the pressure profile above the reversal point is calculated from the standard equation for the slope, using L_3_ as the rise and ϒhk_a_ as the run (a value equal to the lateral earth pressure, expressed this way for the purposes of cancelation). Thus, the slope of the pressure profile below the reversal point can be calculated similarly, using L_4_ as the rise and the product of ϒL_4_k_p_ as the run. Because the slopes are the same, the two equations can be equated. Rearranging to solve for L_3_,

$$ {\displaystyle \begin{array}{l}\frac{\mathrm{L}3}{\varUpsilon \mathrm{hka}}=\frac{\mathrm{L}4\ }{\varUpsilon \mathrm{L}4\mathrm{kp}}\\ {}{L}_3=\frac{hk_a}{k_P}=\frac{\left(6\ \mathrm{ft}\right)(0.4724)}{2.12}\\ {}\kern0.84em =1.337\ \mathrm{ft}\end{array}} $$

The necessary embedment depth is

1.337 ft + 5 ft = 6.337 ft

The total required soldier pile length is

6.337 ft + 6 ft = 12.337 ft (13 ft assumed)

So, I have determined that the solder pile (W12 × 26) should be 13 ft long, and the perpendicular support (w8 × 12) should be 6 ft long as the support for structurally sound maglev construction.

To construct the long lasting and sophisticated underground maglev, I have performed load resistant factor design (LRFD) calculation and selected W24 × 84 beam that the continuous maglev underground runs (structural beam) are structurally sound. Then I have calculated the required shoring concept for 100 miles long and 128 ft wide construction cost considering standard excavation up to 6 ft deep, with appropriate shoring with minimum embedment depth. L4 is 5 ft and standard soil pressure ϒ_s_ = 120 lbf/ft^3^, angle of pressure *Φ* = 21^0^, and the soil pressure coefficient *c* = 800 lbf/ft^2^ in order to determine the length of soldier piles. So, I have calculated by using LRFD methods again that selected that the solder pile (W12 × 26) should be 13 ft long, and the perpendicular support (w8 × 12) should be 6 ft long as the support maglev construction.

## Cost of maglev infrastructure

The proposed maglev infrastructure, therefore, requires shoring, excavation, structural steel, and concrete operation, and thus I have calculated the estimate considering the following components:

Shoring at 13′ deep with w24 × 26 steel soldier piles at 6′ OC both side $2/lf; top rail w8 × 12 both sides $2/lf; 6′ length w8 × 12 perpendicular support 20 OC $2.lf; and protection board 1,372,800 ft^2^ both side at $4/ft^2^ ,and thus the total cost would be $23,724,800.

Excavation (5,2800’_length_ × 128_width_ × 6_deep_ × 1.3_fluff factor_)/27 is 19,524,266.67 yd^3^ at $56/yd^3^ cost for digging, stock piling, and backfilling, and the total cost would be $1,093,358,933.

Cost of materials: 100-mile maglev system with structural steel (w24 × 84) support for eight lanes is $354,816,000; 2 × 2 structural concrete strip footing at $150/yd^3^ is $93,866,666; reinforcement bar at 100 lb./yd^3^ and the cost is $62,577,778; concrete form at $2/ft^2^ is $16,896,000, and thus the total cost of material is $528,156,445.

Cost of labor: 200 iron worker for 2704 working days at $100/h; 100 concrete cement workers for 2704 working days at $90/h; 100 laborer for 2704 working days at $70/h; 50 equipment operator for 2704 working days at $100/h, and thus the total labor cost is $886,912,000 considering standard 8 h a day.

Equipment cost: 10 small renting at $1000/day; 10 small tool renting at $250/day; 271 concrete pump at $2000/each, and thus the total equipment cost is $34,342,000.

Other cost: engineering service at $5/ft^2^; survey team at $4400/day for each working days, and thus the total cost is $349,817,600.

The net construction cost by adding 10% general condition, 10% overhead and profit, and 3% contingency into the excavation, material, labor, equipment, and other cost would be $$3,587,063,487.

## Cost of traditional road infrastructure

A typical highway consists of 8″ asphalt surface course, 4″ binder course, 4″ base course, and 12″ aggregate with standard wiremesh or framing, and thus we have calculated the estimate considering the following components:

Excavation (52,800_length_ × 128_width_ × 2.33_deep_ × 1.3_fluff factor_)/27 is 7,581,924 yd^3^ at $56/yd^3^ cost for digging, stock piling, and backfilling, and the total cost would be $424,587,744.

Cost of materials: $50/yd^3^; 4″ base course is 834,370 yd^3^ at $50/yd^3^; wire-mesh or framing is (528,000 × 128) at $1/ft^2^, and 12″ subbase aggregate is 2,503,111 yd^3^ at $25/yd^3^, and thus the total cost of material is $380,472,775.

Cost of labor: 200 asphalt cement workers for 2704 working days at $100/h; 200 labor foremen for 2704 working days at $100/h; 200 laborers for 2704 working days at $70/h; 200 equipment operators for 2704 working days at $100/h; 100 truck drivers for 2704 working days at $100/h; 200 small roller engineers for 2704 working days at $100/h, and thus the total cost is $2,249,728,000.

Equipment cost: 200 roller renting at $1000/week; 200 milling renting at $10,000/week; 100 truck renting at $500/week, and thus the total cost is $502,171,429.

Other cost: detailing and shop drawing at $10/ft^2^; engineering service at $5/ft^2^; survey team at $4400/day for each working days; banking service of 301,037 yd^3^ at $1000/yd^3^; maiden concrete divider is 106,468 yd^3^ at $818/yd^3^, and thus the total cost is $1,326,694,600.

The net construction cost by adding 10% general condition, 10% overhead and profit, and 3% contingency into the excavation, material, labor, equipment, and other cost would be $6,805,115,863.

## Cost saving

In this article, I have calculated cost saving by using standard 100-mile highway of 128 ft wide (12 ft wide of four lanes on each directions, two-sided 10 ft service space, and 6 ft median in the center of the road) as an experimental tool to compare construction cost in between conventional and maglev infrastructure system. Total cost estimate for traditional infrastructure is $6,805,115,863, and the maglev infrastructure system cost is only $3,587,063,487 for the same 100-mile highways and the net cost saving is $3,218,052,377 (Table 1). Consequently, it will reduce neatly 50% of the cost once maglev infrastructure system is used for the construction of invisible infrastructure which is also benign to the environment.

**Table 1:**
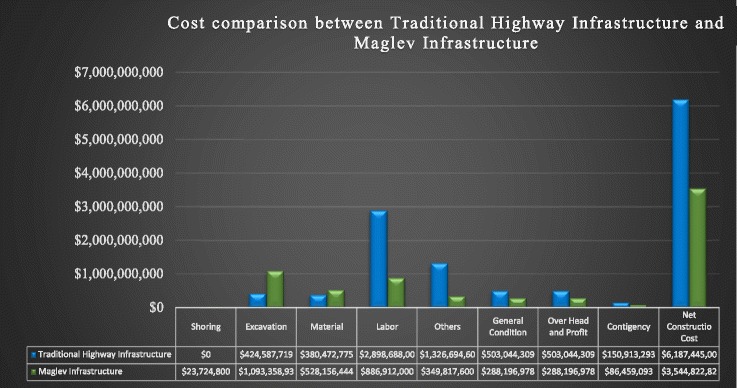
This cost comparison is prepared by using HCSS cost data 2016 for material by utilizing selective manufacturers and labor rate in accordance with international of union wage of each specified trade workers considering US location. The equipment rental cost is estimated as current rental market in conjunction with the standard practice of construction of the production

## Conclusions

Traditional transportation infrastructure construction throughout the world is not only expensive, but also consumes 5.6 × 10^20^ J/yr (560 EJ/yr) fossil fuel each year which indeed dangerous of a cliché when discussing about climate [33, 34]. To mitigate these problems, better infrastructure transportation planning is needed to be done where environmental sustainability and climate adaptation are to be confirmed for the creation of communities more resilient and vibrant. Interestingly, the *Maglev Infrastructure Transportation* technology proposed in this article, for urban infrastructure transportation system, implicated by electromagnetic and superconducting magnets will, thus, be the emergent technology in modern science to console infrastructure, energy, and environmental dire straits, just because this technology is cheaper and will run by repulsive-force and attractive-force at the levitated (flying) stage while it will run on maglev system and will run by air (wind energy) while it is on non-levitated area without consuming fossil fuel. Indeed, this new maglev infrastructure transportation system would be the innovative technology ever to console infrastructure, transportation, energy, and global warming crisis.
